# CaPTure: Calcium PeakToolbox for analysis of in vitro calcium imaging data

**DOI:** 10.1186/s12868-022-00751-7

**Published:** 2022-11-30

**Authors:** Madhavi Tippani, Elizabeth A. Pattie, Brittany A. Davis, Claudia V. Nguyen, Yanhong Wang, Srinidhi Rao Sripathy, Brady J. Maher, Keri Martinowich, Andrew E. Jaffe, Stephanie Cerceo Page

**Affiliations:** 1 Lieber Institute for Brain Development, Johns Hopkins Medical Campus, 855 North Wolfe Street, Suite 300, Baltimore, MD 21205 USA; 2 Department of Psychiatry and Behavioral Sciences, Johns Hopkins School of Medicine, Baltimore, MD 21205 USA; 3 Department of Neuroscience, Johns Hopkins School of Medicine, Baltimore, MD 21205 USA; 4 McKusick-Nathans Institute, Department of Genetic Medicine, Johns Hopkins University School of Medicine, Baltimore, MD 21205 USA; 5 Department of Biostatistics, Johns Hopkins Bloomberg School of Public Health, Baltimore, MD 21205 USA; 6 Department of Mental Health, Johns Hopkins Bloomberg School of Public Health, Baltimore, MD 21205 USA

**Keywords:** Calcium imaging, hiPSC, Neuronal activity, Image analysis

## Abstract

**Background:**

Calcium imaging is a powerful technique for recording cellular activity across large populations of neurons. However, analysis methods capable of single-cell resolution in cultured neurons, especially for cultures derived from human induced pluripotent stem cells (hiPSCs), are lacking. Existing methods lack scalability to accommodate high-throughput comparisons between multiple lines, across developmental timepoints, or across pharmacological manipulations.

**Results:**

To address this need we developed CaPTure, a scalable, automated Ca^2+^ imaging analysis pipeline (https://github.com/LieberInstitute/CaPTure). CaPTuredetects neurons, classifies and quantifies spontaneous activity, quantifies synchrony metrics, and generates cell- and network-specific metrics that facilitate phenotypic discovery. The method is compatible with parallel processing on computing clusters without requiring significant user input or parameter modification.

**Conclusion:**

CaPTure allows for rapid assessment of neuronal activity in cultured cells at cellular resolution, rendering it amenable to high-throughput screening and phenotypic discovery. The platform can be applied to both human- and rodent-derived neurons and is compatible with many imaging systems.

**Supplementary Information:**

The online version contains supplementary material available at 10.1186/s12868-022-00751-7.

## Background

Because transient dynamic changes in intracellular calcium concentration occur rapidly during the course of neuronal activity, calcium imaging is frequently employed for assessing neuronal activity. Measurements of intracellular calcium levels can be used to quantify cellular activity at both network-wide and single-cell resolution. In vivo two-photon microscopy via thin-skull or cranial window preparations has been employed for over a decade to perform calcium imaging in head-fixed rodents, and more recently used to measure activity dynamics in the brains of awake, behaving animals using fiber photometry or miniaturized microscopes coupled with endoscopic imaging. Advances in methodology and technology have rapidly increased the experimental capabilities of calcium imaging (as recently reviewed [1–3]), and with this have emerged a number of computational methods to analyze calcium imaging data both at the level of bulk calcium dynamics and in single cells [4–8].

However, due to differences in signal-to-noise ratios and background fluorescence in intact tissue versus cell culture systems, collecting and analyzing calcium imaging data from in vitro cell culture models requires different computational approaches. For example, in vitro cell model systems are comparatively less active and more synchronous than intact brain samples. Many of the existing methods for calcium imaging analysis detect changes in activity, and then combine those synchronous signals into the signal attributed to a single cell [6, 9]. Due to the high degree of synchronicity in in vitro systems, these methods erroneously combine activity measurements for multiple cells that are firing as an ensemble. With advancements in human induced pluripotent stem cell (hiPSC) technologies and in vitro genetic modeling of disease, the need to accurately measure neuronal activity in cultured neurons is increasingly important. As current models often involve either co-culture systems with multiple species as source material (e.g. rodent glial cells co-cultured with human neurons) or mixed cell-type assemblages (e.g. primary cortical tissue, or hiPSC-derived organoids), genetically encoded calcium indicators (GECIs) enable important cell-type specific targeting. Thus, strategies for measuring neuronal activity that use AM-dye based Ca^2+^ indicators or multi-electrode arrays, where a priori targeting or characterization of a specific cell population is not feasible, result in limited cell-type specific information.

Acquisition of this information enables comparisons between hiPSC lines derived from different individual donors, or from transgenic rodent models. Reviewing the existing literature, we found that most analysis methods, e.g. *‘findpeaks’* in MATLAB, require a high degree of user input to define parameters [10, 11], or extensive knowledge of the data being acquired to provide information for specific functions. On the other hand, FluoroSNNAP—Fluorescence Single Neuron and Network Analysis Package—accurately detects events, but is GUI based and thus is not compatible with high performance computing clusters [12]. Utilizing a field-based thresholding approach requires a high degree of similarity between all acquired time-lapse movies, or the selection of amplitude and intensity thresholds to be performed for each field independently, which is not scalable for large datasets.

Here we introduce CaPTure, which is an automated analysis pipeline that facilitates (1) the accurate detection of neurons, (2) the identification of calcium events in individual cells, and (3) the calculation of image-based network connectivity metrics. Utilizing a construct that expresses both a fluorescent cellular label and GECI in the cell type of interest, we extended the FluoroSNNAP software package by introducing additional data pre-processing steps to detect regions of interest (ROIs) to focus subsequent analysis, and normalize fluorescence intensity over time [12]. We added data-driven motifs representing events observed in our data, and calculated synchrony metrics including clusters of synchronous cells to assess ensemble activity. We demonstrate that our method accurately quantifies dynamic measurements in selected cells, while incorporating both per-field and individual per-ROI neuronal activity metrics. Thus, this method has the advantage of facilitating comparisons of neuronal and network activity between genetic models of disease and pharmacological manipulations. The method is highly amenable to parallel computing and high-throughput screening.

## Implementation

### Sample preparation

#### hiPSC-derived neurons

Fibroblast donors were male and of European ancestry—these research subjects were enrolled in the Sibling Study of Schizophrenia at the National Institute of Mental Health in the Clinical Brain Disorders Branch (NIMH, protocol 95M0150, NCT00001486, Annual Report number: ZIA MH002942053, DRW PI) as previously described [13]. Early passage fibroblasts (< 5 passages) were reprogrammed into hiPSCs as previously described [14], and subsequently differentiated through neural progenitor stages into cortical neurons. Neurons were co-cultured in 24-well ibidi plates (Cat. No. 82406, ibidi GmbH, Munich, Germany) with astrocytes prepared from the cortices of neonatal rats to promote neuronal maturity as previously described [13, 15]; and were maintained with partial media changes twice a week for up to 10 weeks (Day in Vitro (DIV70)).

#### Animals

Timed-pregnant Wistar rats for astrocyte cultures were obtained from Charles River Laboratories (Wilmington, MA, USA; stock Crl:WI003). To obtain fetal tissue, pregnant dams were euthanized by carbon dioxide asphyxiation followed by cervical dislocation. Mice were purchased from Jackson laboratories (Bar Harbor, ME, C57BL6/J; stock #000,664), and bred for the generation of postnatal day 0 mice primary neuronal cultures. To obtain neonatal tissue, pups were anesthetized by being placed on ice, followed by rapid decapitation, and dams were returned to the breeding colony. All rodents were housed in a temperature-controlled environment with a 12:12 light/dark cycle and ad libitum access to standard laboratory chow and water.

#### Mouse primary cortical cultures

Mouse cortical neurons were cultured as previously described with modifications [16]. Briefly, on the day of birth, mice were anesthetized by being placed on ice, then rapidly decapitated and their cortices removed. Cortical tissue was dissociated using papain, and plated at a density of 2.5 × 10^5 per well on a 24-well ibidi plate (Cat. No. 82406, ibidi GmbH, Munich, Germany) coated with poly-D-lysine and laminin. Neurons were maintained in culture with partial media changes every 2 days, and imaged between DIV14 and DIV15.

#### Viral transduction

hiPSC-derived neurons were transduced at DIV23 with adeno-associated virus expressing mRuby2 and GCaMP6s under the control of a synapsin promoter (MOI ~ 6 × 10^4, Addgene viral prep # 50,942-AAV1 [17]. Following a full media exchange on DIV25, neurons were cultured for at least 21 days and imaged on DIV 42 or 63. Mouse primary cultures were transduced with 1:10 viral concentration used in human experiments of the same virus (human synapsin 1 promoter was ubiquitously expressed in mouse neurons). Mouse primary cultures were infected at DIV5–DIV8 prior to DIV14–DIV15 recordings.

### Image acquisition

#### LSM780 confocal microscope

Primary mouse cortical cultures and hiPSC-derived neurons were imaged in culture media on a Zeiss LSM780 equipped with a 10X/0.45NA objective, a temperature- and atmospheric-controlled enclosure to maintain neurons at 37° and 5% CO_2_. A reference image was acquired for each field of mRuby fluorescence followed by a time-series was acquired at 4 Hz for 8 min. In some cases, tetrodotoxin (TTX, 1uM) was then added to block synaptic transmission and incubated for at least 5 min prior to imaging to equilibrate.

#### Spinning disk confocal microscope

Neurons were removed from culture media and were continuously perfused with artificial cerebro-spinal fluid (ACSF) containing (in mM): 128 NaCl, 30 glucose, 25 HEPES, 5 KCl, 2 CaCl_2_, and 1 MgCl_2_ (pH 7.3) [15]. Imaging was performed at DIV56 or DIV70 on a custom-built Zeiss AxioExaminer Z.1 equipped with a live-slice Yokogawa spinning disk module, Flash4.0 V3 sCMOS camera, and a 20X/1.0NA water immersion objective. A reference image was acquired using mRuby fluorescence, then a time-series was acquired at 10 Hz for 5 min. For experiments in which pharmacological blockers were added, TTX (1 uM) was included in the perfusate for at least 5 min prior to imaging.

#### Acquisition parameters

From all microscopes, two image types are collected: a time-series of GCaMP6s fluorescence and a reference image of mRuby to demarcate infected neurons. The reference image of the LSM780 scope is downsampled using the MATLAB function *imresize* to match the time-series image in X and Y dimensions.

**Table:** 

Scope	Reference image X Y	Pixel to micron	Time-series image X Y	Pixel to micron
LSM 780	1024 × 1024 pixel	0.83 × 0.83 μm per pixel	256 × 256 pixel	3.32 × 3.32 μm per pixel
Spinning disk	1024 × 640 pixel	0.645 × 0.645 μm per pixel	1024 × 640 pixel	0.645 × 0.645 μm per pixel

#### Toolbox installation and software requirements

All data processing for CaPTure is conducted in MATLAB (Version 2017a or later). The processing pipeline is divided into several steps as described below, the execution of which are explained in the following repository https://github.com/LieberInstitute/CaImg_cellcultures. The repository consists of a `toolbox` directory whose path needs to be added to the MATLAB working directory to run any of the processing steps. The directions to download and install the toolbox are described in the *‘installation’* step of the repository.

#### Statistics

To calculate the effect of pharmacological manipulations, the lmerTest R package [18] was used for performing linear mixed effects modeling as a function of treatment main effect (Baseline versus TTX) and used cell line and the cell culture experimenter as the random intercepts to control for variability in the cell culturing process.

### Using CaPTure

In this section we describe the analysis workflow: first we identify ROIs by segmenting neurons in the cell-fill channel, and then extract fluorescence intensity. Then we identify "peaks,” which are used to calculate per-image and per-cell summary and aggregate metrics to assess network and cellular activity.

#### Step1: Convert .czi time series files to.mat files

A time-series of images was collected for each imaging field, and saved using the Zeiss proprietary.czi file format to maintain image metadata. Since all of the image data processing is performed in MATLAB, we recommend that users convert the raw data to MATLAB format for fast and easy access. We use the Bio-Formats package called `bfmatlab` [19] to load the*. czi* data into MATLAB and the MATLAB save function to save it to *.mat* format. The `bfmatlab` package supports the conversion of multiple proprietary file formats obtained from different microscope systems, thus enabling the use of CaPTure on calcium imaging data obtained from various systems.

#### Step2: Identify ROIs

**Fig. 1:**
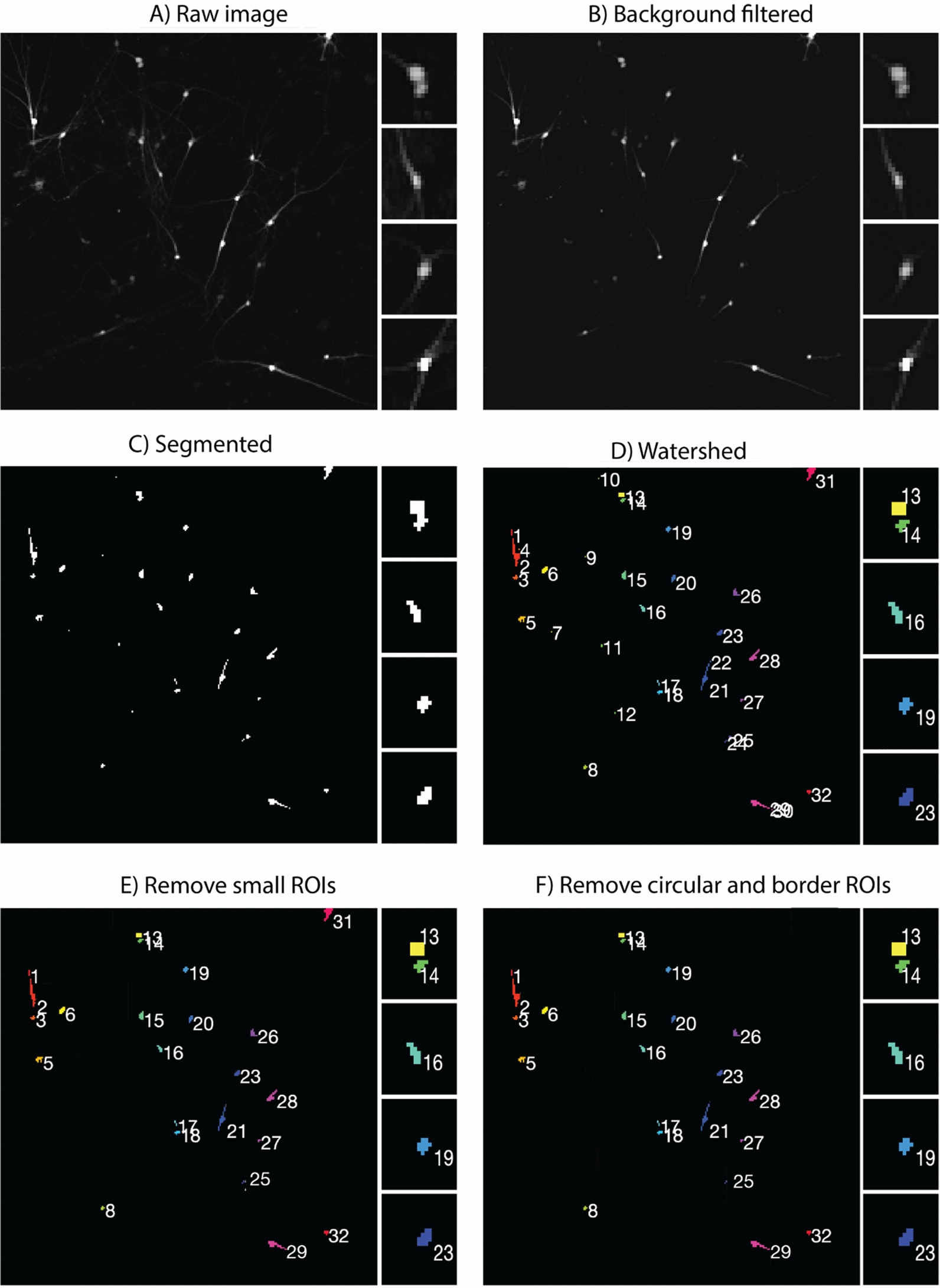
Nuclei Segmentation: **A** Raw *‘.czi’* image of neuronal cultures. **B** Background filtered image of the raw *‘.czi’* image. We used the MATLAB function *‘imhmin’* (with 2 times the standard deviation of image as threshold) to suppress the signal derived from the neurites. **C** Segmented (using *‘Region Growing’*) binary image of neuronal cell bodies. **D** Final watershed segmentation of ROIs from the binary image (inserts of ROI 13 and 14). Watershed was performed for better extraction of individual ROIs that are spatially in close proximity. We adjusted parameters (ROI size and eccentricity of ROI) to ensure that unintended splits and mergers were minimal. **E** ROIs (e.g., 4, 7, 11) with total pixel sizes below the size threshold are excluded from the final segmentation. **F** ROIs with an eccentricity > 0.99, indicating a line, and ROIs on the image border (i.e., 31) are excluded

CaPTure allows the user to automate detection of ROIs, and then to select ROIs based on their shape or size. The strategy allows us to detect cells that express the cell-type specific GECI, but are inactive. From each reference image, we identify infected neurons from which to measure calcium dynamics (Fig. 1A). Neurons have a complex morphology, and we aimed to identify signal from the soma, and not from surrounding neuropil. Thus, we used the MATLAB function *‘imhmin’* to suppress the background signal coming from the neurites (Fig. 1B). We then used the *region growing* technique [20] for segmenting ROIs from the red image, where the pixel with the minimum fluorescence intensity of the image is chosen as the initial seed location, and the region is iteratively grown by comparing all unallocated neighboring pixels to the seed region. The difference between the intensity value of each pixel and the mean of the region is used as a measure of similarity. The pixel with the smallest difference measured this way is allocated to the respective region. This process stops when the intensity difference between the region mean and that of the new pixel becomes larger than a user specified threshold, in this case, the standard deviation of the image (Fig. 1C). The fully grown region is termed the background, thus leaving out the regions with high intensity which become the final segmented ROIs (Fig. 1D). To select for neurons and to remove noise, debris and neuropil from further inclusion in the data, we used eccentricity (a measure of the roundness of the ROI calculated by the MATLAB function *‘regionprops3’*) and a minimum size threshold to filter out ROIs from neuropil and noise (Fig. 1E, F). The output of Step 2 provides the identification of all ROIs. The output of alternative segmentation algorithms [21] can be integrated and used for extraction of downstream activity traces.

#### Step3: Extract traces from each ROI

**Fig. 2:**
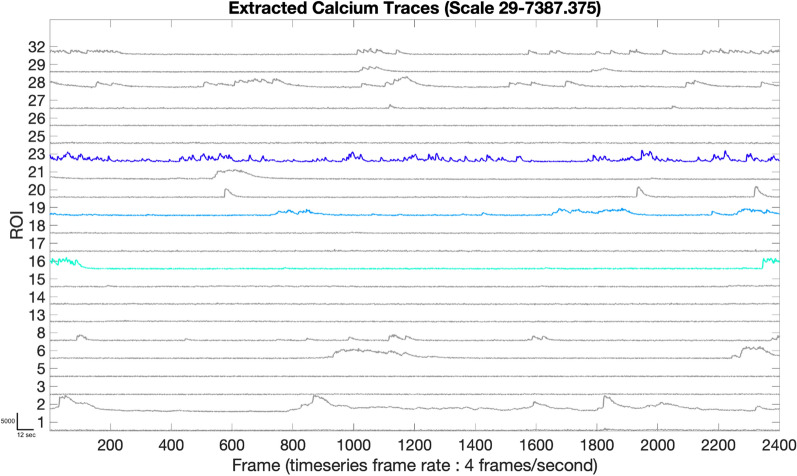
Extracted calcium traces: the graph shows the calcium activity for each ROI segmented in Step2, and highlights traces 16, 19, and 23 as examples of low-, medium- and high-activity ROIs, respectively. The x-axis is the frame number of the time series and the y-axis range reflects the minimum and maximum fluorescence intensity of the ROIs in this image series (29 and 7387 units of mean fluorescence intensity, respectively, in this example trace). Scale bar indicates: 12 s on x-axis and 5000 units of mean fluorescence intensity on y-axis

Calcium imaging allows for measurement of calcium levels in each individual cell by measuring dynamic fluorescence intensity. From each identified neuron, i.e., ROI, we extract calcium signals by measuring the fluorescence intensity over time. Traces (signal) are extracted from the green video using the ROI segmentations from Step 2. Each point on the trace is the average intensity of all the pixels of the segmented ROI at that Z frame in the green video. The output of Step 3 (Fig. 2) is raw traces for each ROI. For ease of illustration, in subsequent figures we focus on three ROIs: ROI 16-low activity (light teal), ROI 19-moderate activity (medium blue) and ROI 23-high activity (royal blue).

#### Step4: Extract delta fluorescence/fluorescence (dff) from step3

**Fig. 3:**
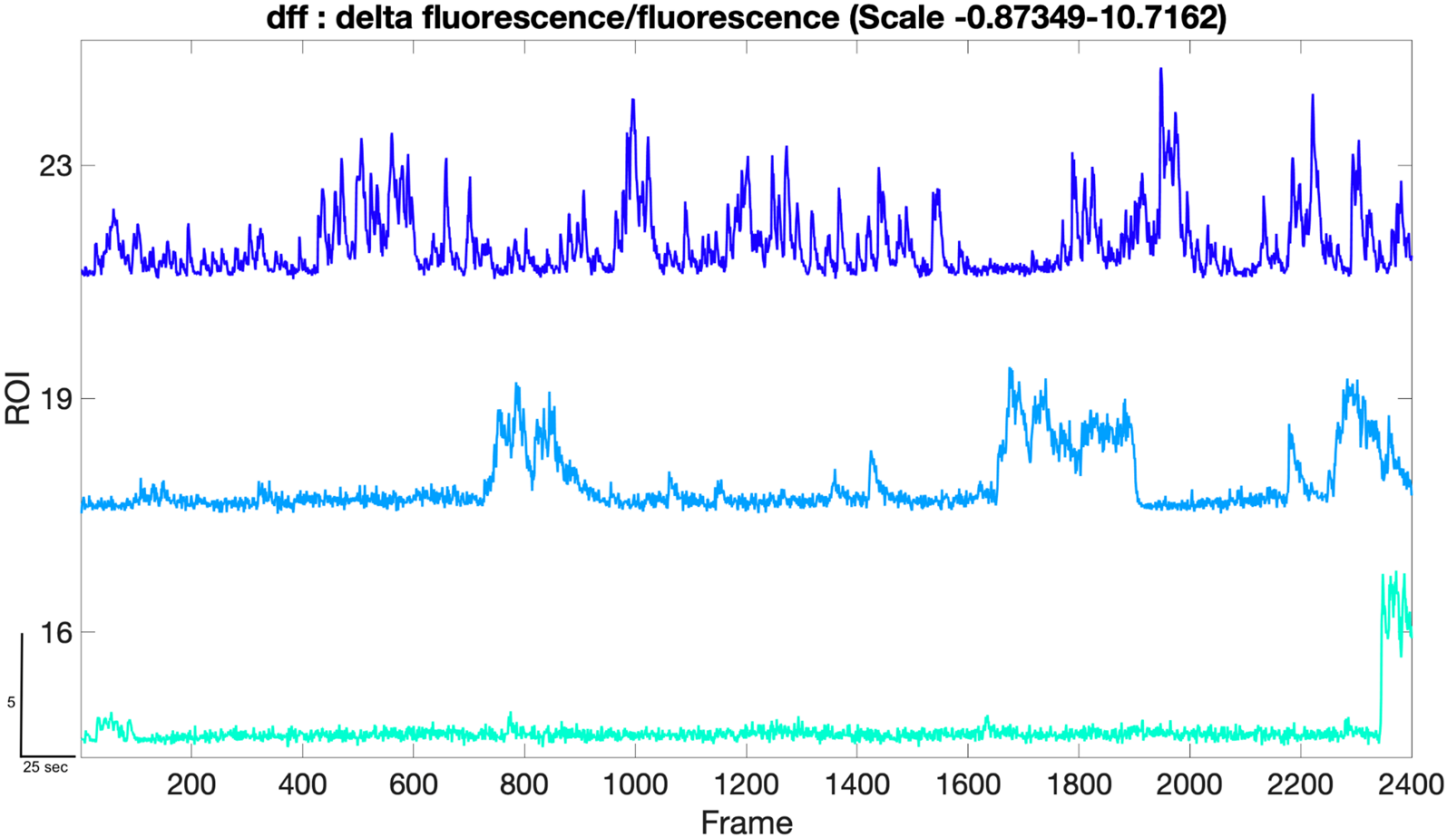
Delta fluorescence/fluorescence: the graph shows the normalized calcium traces extracted from the calcium activity shown in Fig. 2. The x-axis is the frame number of the time series and the y-axis range reflects the normalized minimum and maximum fluorescence intensity (− 0.87349 and 10.7162) of the ROIs 16, 19 and 23 which shows low, medium and high activity. Scale bar indicates: 25 s on x-axis and 5 units of normalized mean fluorescence intensity on y-axis

Due to fluctuations in viral transfection efficiency, baseline activity, expression of the virus and the position of the cell within the sample, there can be differences in the baseline fluorescence intensity fluctuations between ROIs. We thus normalized dynamic fluorescent intensity to baseline by calculating the change in fluorescence using a rolling average [22] to obtain the DF/F following standard methodology. The output of Step 4 provides normalized traces with smoothing (Fig. 3).

#### Step5: Construction of correlation maps

**Fig. 4:**
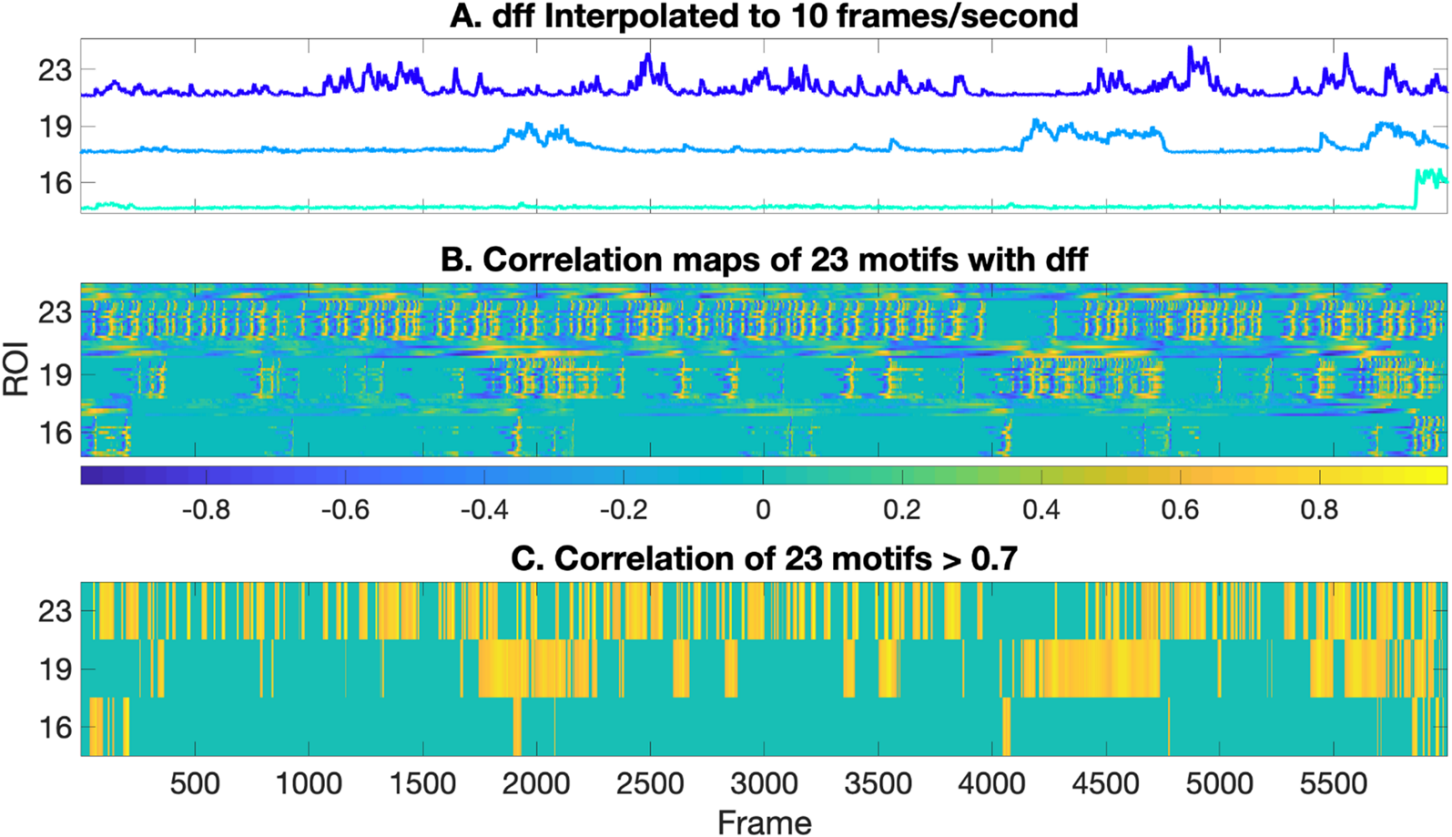
Motif correlation maps: **A** the normalized traces (4 frames/sec) from step4 are interpolated to 10 frames/sec to match the frame rate of the motifs being correlated. **B** Motif correlation map showing frames in yellow when the event predominantly matches a motif, and frames in blue when the event is less matched with the same motif. The frames in turquoise represent the background. **C** The correlation matrix is thresholded (threshold = 0.7). Frames where the maximum correlation (of 23 motifs) is above the threshold are shown as yellow

To identify calcium events, a correlation map is constructed to compare the pattern of fluorescence intensity changes with known motifs representing calcium events. Prior to the calculation of the correlation map, the dff traces needed to be interpolated because the motif library, created by FluoroSNNAP [12], utilized a frame rate of 10 frames/sec (Fig. 4A). We utilized the FluoroSNNAP motifs (Additional file 1: Figure S1: 1–16) and constructed seven motifs (Additional file 1: Figure S1: 17–23) based on observations from our data. A matrix (‘Ca’, rows = motifs, columns = x axis of the trace) of correlation coefficients of all motifs across the trace is computed (Fig. 4B). The correlation coefficients are set to a value of zero at locations across the trace where the intensity/height of the trace are below a certain threshold that represents the background, to avoid noise (Fig. 4C). The output of Step 5 aligns normalized traces to motifs (Fig. 4).

#### Step6: Extract event location and duration

**Fig. 5:**
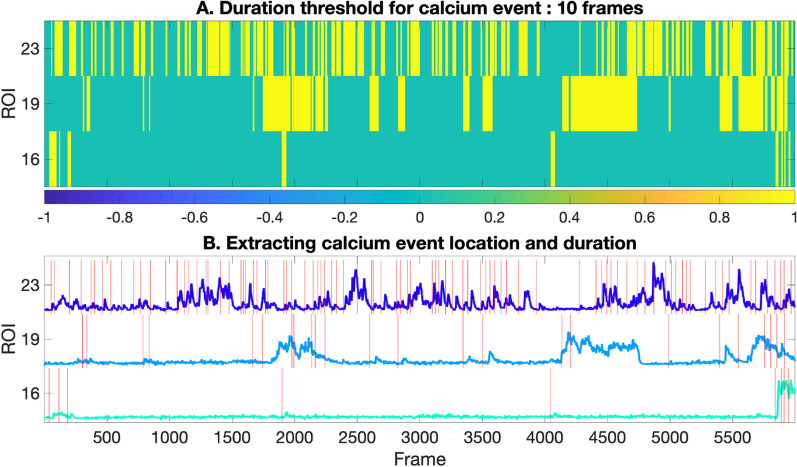
Extract calcium events: **A** the thresholded correlation map from Fig. 4 is converted to a binary map, in which events are colored as yellow, while background is teal. **B** We then displayed the xtracted event location and duration based on the binary map in **A** on the dff traces from Step2

**Fig. 6:**
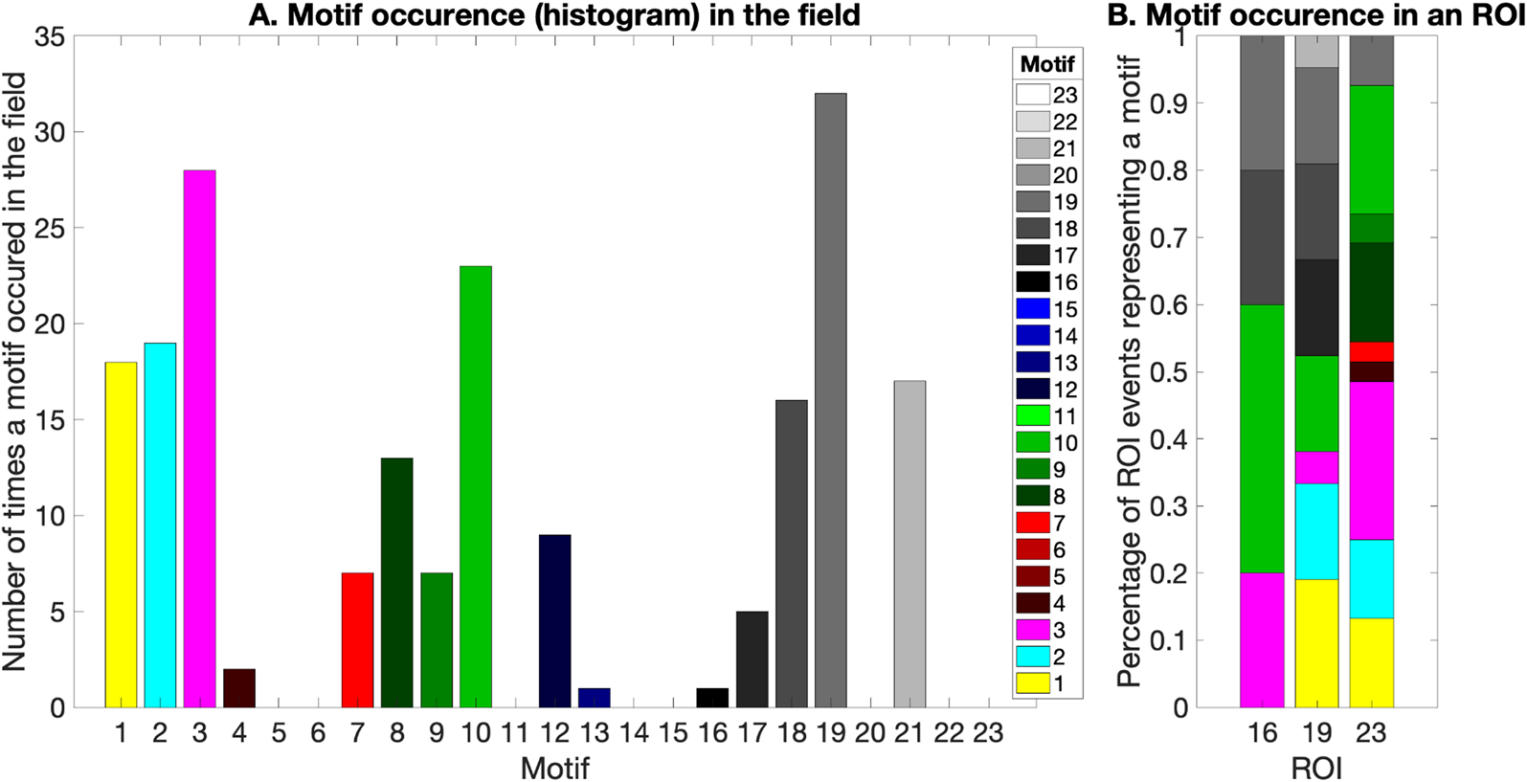
Frequency of a motif occurrence: **A** the barplot shows the frequency of occurrence of a motif in the specific field. The x-axis shows individual motif and the y-axis shows the total number of times the motif appeared in the field. **B** The barplot shows the percentage of events in a ROI that correlates with a specific motif. The x-axis shows the ROIs 16, 19, 23 and the y-axis shows the percentage of events of the ROI

We next extract the event location and duration for each event in each ROI (Fig. 5). A final row matrix is computed by picking the maximum correlation coefficient from each column of ‘Ca’. The points that exceed the user given correlation threshold (0–1) on the row matrix represent the events of that trace. A high correlation threshold might result in missing some events, while a low correlation threshold will potentially pick noise as events, so an optimal threshold of ~ 0.7 was used for our datasets (Fig. 5B). The total number of all the consecutive points/frames that cross the threshold is taken as the event duration in frames. The output of Step 6 counts and classifies motifs (Fig. 5). We illustrate the occurrence of each motif in our example data set (Fig. 6A), and the occurrence of each motif within each ROI (Fig. 6B).

#### Step 6A (optional): Synchronicity

**Fig. 7:**
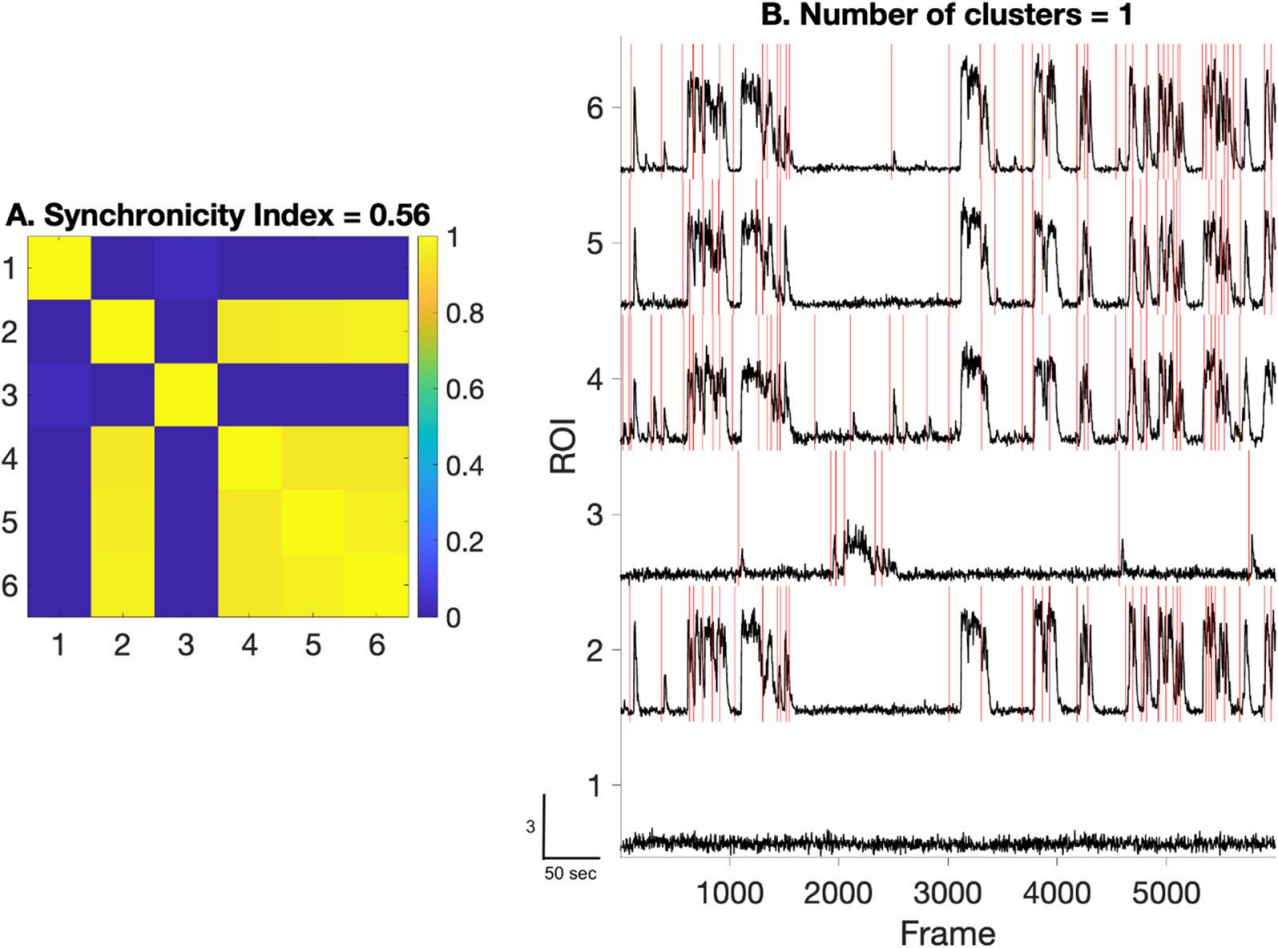
Network synchronicity: **A** heatmap showing pairwise correlation of the calcium activity in the field. Synchronicity Index (0–1) represents a measure for network synchrony of the field. **B** showing calcium activity of the field to visually analyze network synchronicity. Scale bar indicates: 50 s on x-axis and 3 units of normalized mean fluorescence intensity on y-axis

Because neurons in in vitro networks are highly interconnected, we aimed to estimate the degree to which calcium events were synchronous across a given field. To do this we quantified how synchronous the calcium activity is between the ROIs of a given field using the functions (*‘SCA’*) provided by the FluoroSNNAP package (Fig. 7). The package provides different methods to quantify synchrony including phase correlation, entropy, and Fourier Transforms of the calcium traces and events. We used the correlation method applied on calcium activity and corresponding surrogate traces of pairwise neurons in a field to quantify the network synchronicity [12]. Eigenvalue decomposition is used on the pairwise correlation matrix of the ROIs, which decomposes the matrix into clusters of ROIs with similar activity and quantifies the synchronization of each ROI cluster. The output of Step 6 shows the degree to which events in each ROI are correlated with events in other ROIs.

#### Step7: Extract final data

A custom MATLAB script was written to extract two types of metrics: individual ROI metrics in the file *long_dat* and image metrics in the file *man*. This allows us to make comparisons across individual cells and across fields. The final *man.csv* file represents the image level summary statistics (in columns) for each image (in rows) in the dataset.

**Table:** 

Name	Image name
Metadata	Biological and metadata associated with the image
num_ROI	Number of cells identified in the red image
num_active_ROI	Number of cells that fire at least one calcium event
prop_active_ROI	Proportion of active cells in the image
corrSYN	Synchronicity index describes how synchronous are the cells in the image in firing events
motif(1–23)	Frequency of occurrence of each motif in the time series of the all the ROIs in the image

The final *long_data.csv* file represents the ROI level summary statistics (in columns) for each ROI (in row) in the dataset.

**Table:** 

Name	Image name which the ROI belongs to
Metadata	Biological and metadata associated with the image
events_ROI	Number of calcium events that a cell produced
avg_width	Average duration (frames) of events for that ROI
Volume	Number of pixels in red image that corresponds to the ROI
Eccentricity	Describes if the ROI is more elongated or more circular in shape. An ROI whose eccentricity is 0 is actually a circle, while an ROI whose eccentricity is 1 is a line segment
motif(1–23)	Frequency of occurrence of each motif in the time series of the ROI

## Results

To demonstrate the utility of the workflow, we apply CaPTure to several in vitro preparations of neurons (e.g. mouse primary cortical neurons and hiPSC-derived neurons), and demonstrate versatility by applying the workflow to data acquired on an additional microscope system. Finally, we demonstrate the robustness of the method by blocking neuronal activity in iPSC-derived neurons with pharmacological agents and assessing algorithm performance.

**Fig. 8:**
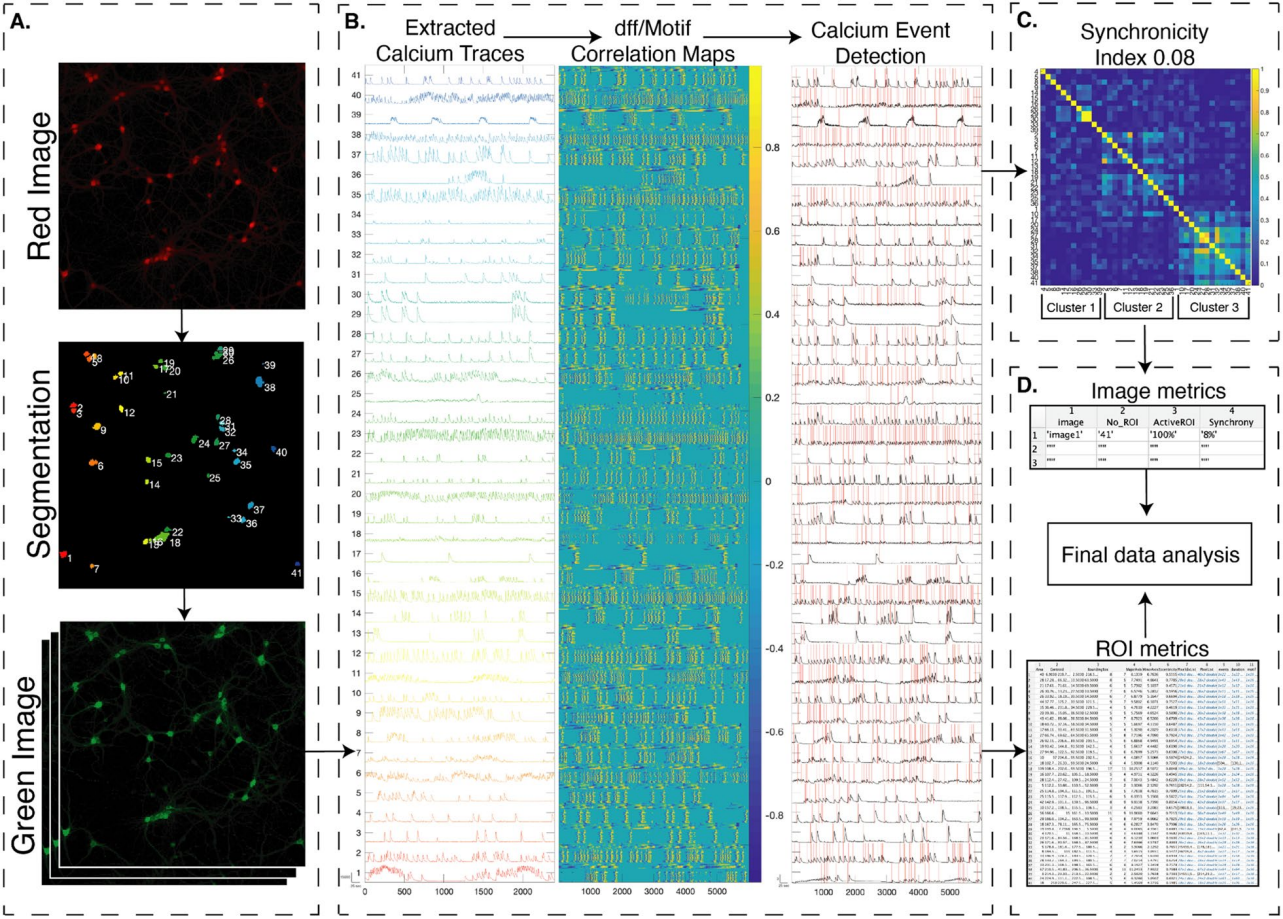
Primary mouse cortical neuronal culture data processed through CaPTure:** A** the raw fluorescent image of mRuby-expressing neurons, its corresponding color-coded neuronal segmentation and the GCaMP6s time-series. **B** Extracted raw calcium traces, the motif correlation map of the interpolated dff traces and event detection on the interpolated dff traces using the motif correlation maps. Scale bar indicates: 25 s on x-axis and y-axis is 15000 units of mean fluorescence intensity for raw traces (column 1), 3 units of normalized mean fluorescence intensity for normalized traces (column 3). **C** Correlation map of inter-neuron calcium activity grouped by the cluster. **D** Final extracted metrics at image (field) level and ROI (neuron) level

We first applied this toolbox to mouse cortical neurons in culture. These cultures are both more dense and more mature than hiPSC-derived neuronal cultures. We confirmed that our ROI detection method accurately identified ROIs and extracted calcium events in an active, dense mouse culture system (Table 1, Fig. 8A, B). We identified unique patterns of synchronicity, which suggests that some sets of neurons preferentially fire together (Fig. 8C). We then extract image- and ROI-based metrics for final data analysis (Fig. 8D).

**Table 1:** Summary of data extracted from two in vitro data sets: human iPS-derived neuronal cultures and mouse cortical neuron cultures.

Sample	Cells	ROIs	Active ROIs	Avg field activity/ 60 secs	Synchronicity	Clusters
Figure 1	Human	22	16	0.9 events/ROI	0.03	4
Figure 8	Mouse	41	41	3.48 events/ROI	0.08	3

**Fig. 9:**
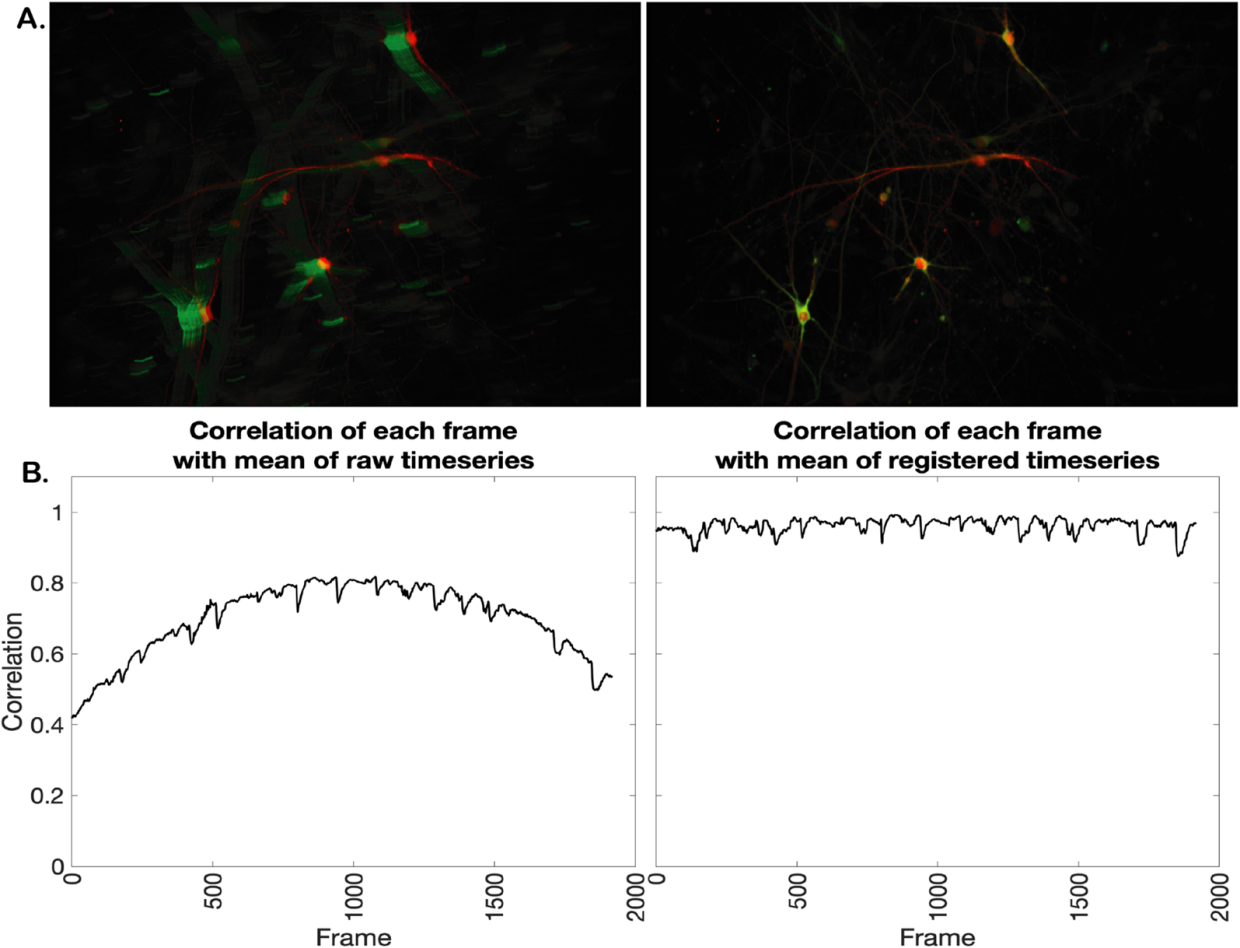
Image registration to correct physical drift: **A** Red image overlaid on the maximum intensity projection of the green time series of the raw data (left) and the registered data (right). **B** Graphs showing correlation (y-axis) of each frame (x-axis) with the mean intensity image of the time series for raw data (left) and registered data (right)

**Fig. 10:**
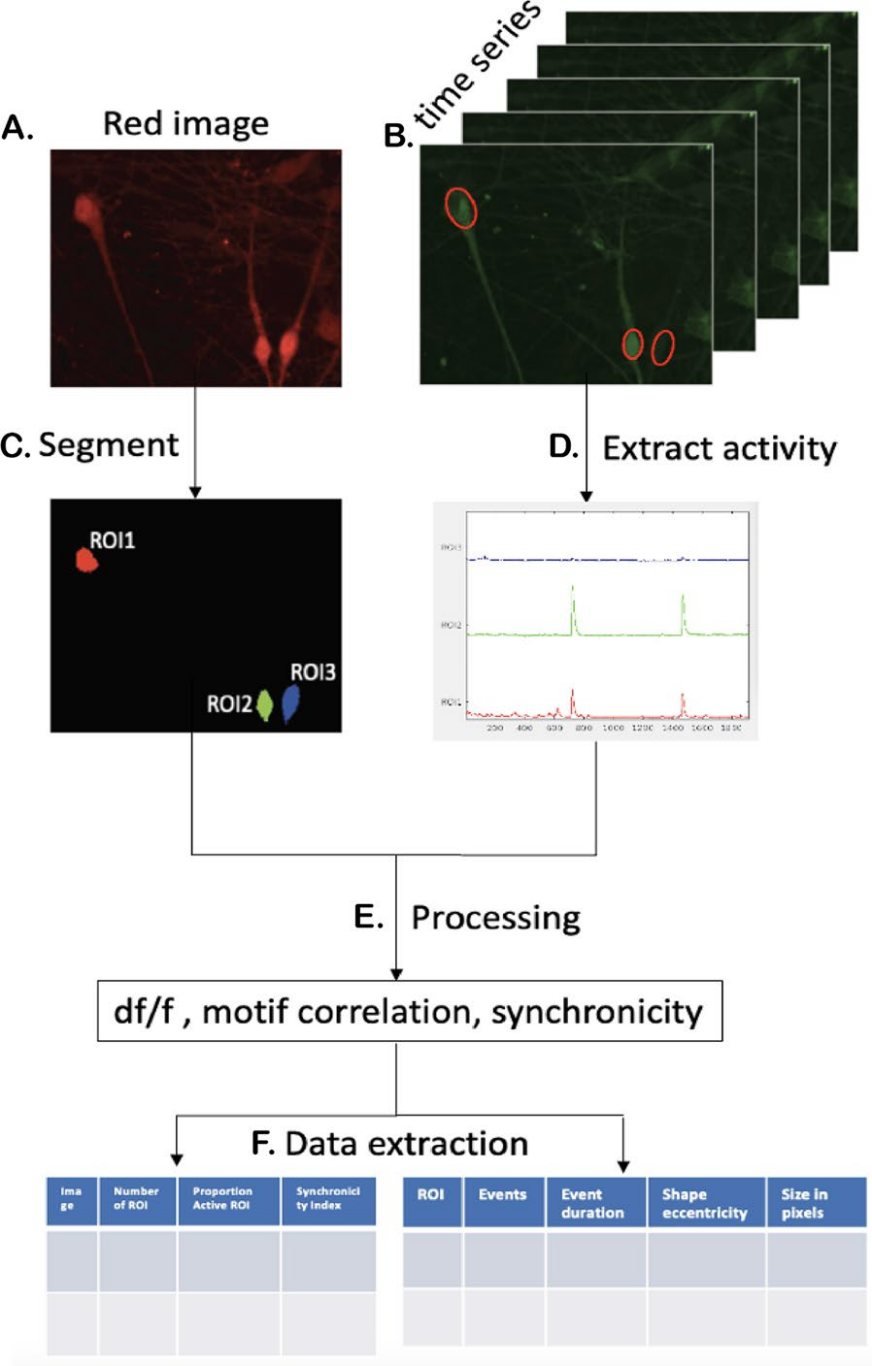
CaPTure applied to secondary data set: **A** Raw *‘.czi’* image of nuclei and **B** the corresponding raw *‘.czi’* time series. **C** Segmentation of nuclei image. **D** Calcium activity extracted from times series for each segmented nuclei. **E** Different processing steps used in CaPTure. **F** Final metrics extracted into tables for image level and ROI level data

Additionally, we applied CaPTure to images acquired on a higher-resolution microscope with a smaller field of view. An additional challenge with this data set was the presence of drift over the time series. Unlike in vivo calcium imaging data, in which movement of the active neuron population is generally due to movement of the animal [23], in this case, the continuous perfusion of ACSF over the coverslip containing neurons resulted in the movement of the sample and thus, the need for rigid registration of images. Since the drift was physical and of known direction, and no landmarks, such as vasculature, are present in cultured neurons, we registered the images by aligning each frame iteratively to the preceding frame, and then aligning the green GCaMP6s time-lapse to the red cell-fill image (Fig. 9A, [24]). To demonstrate the utility of this approach, we show the correlation of the fluorescence of each frame with the mean of the entire time series (Fig. 9B). Prior to registration, the mean of the time series is not highly correlated, and the correlation is variable by frame. After registration, correlation of each frame is highly correlated. Following registration, we applied CaPTure to this set of data, identifying ROIs and individual peaks (Fig. 10).

**Fig. 11:**
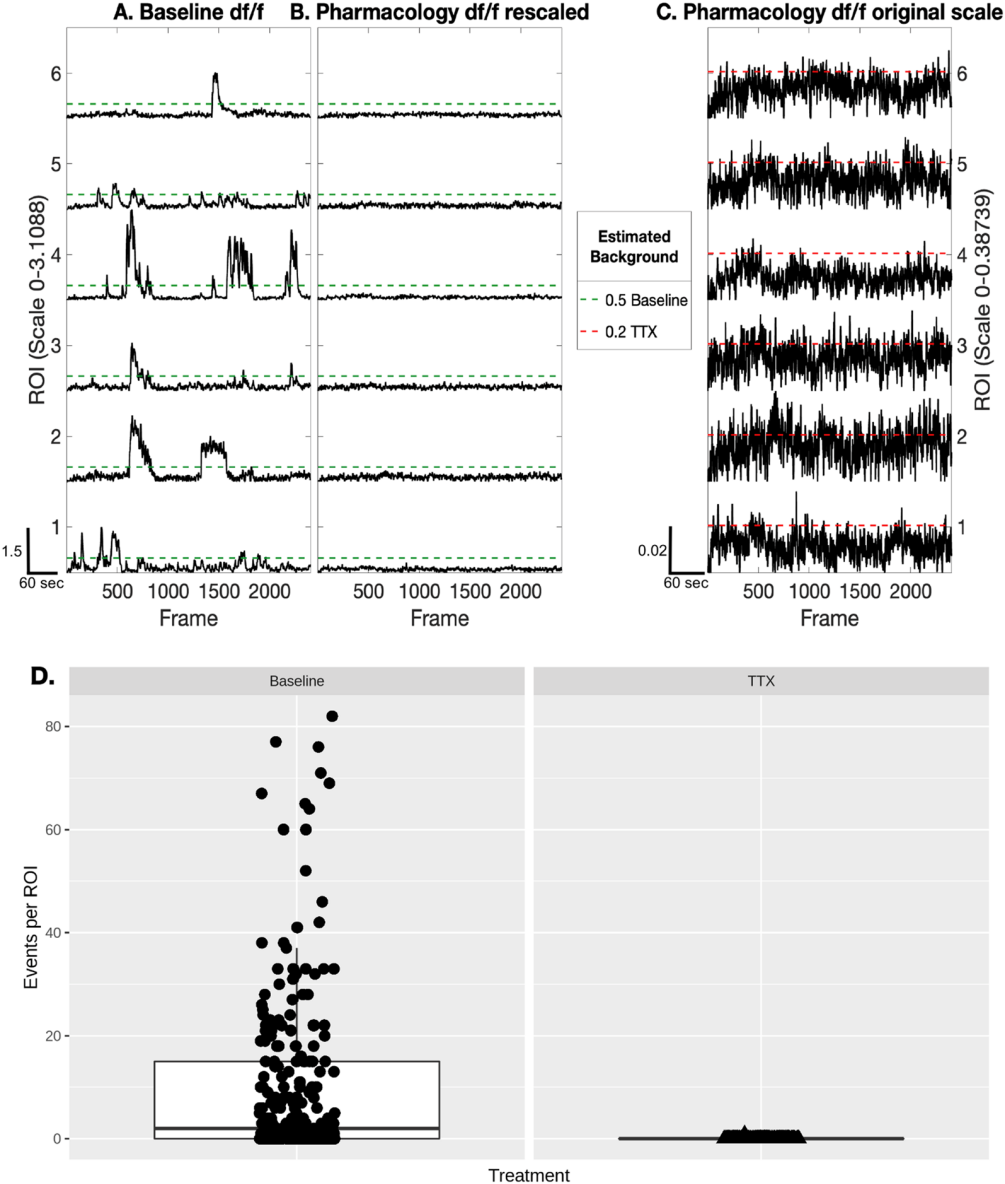
Pharmacological blockade of synaptic transmission illustrates the specificity of CaPTure: **A** graph showing normalized calcium activity from a sample baseline field and the estimated background (0.5), Scale 0–3.1088. Scale bar indicates: 60 s on x-axis and 1.5 units of normalized mean fluorescence intensity on y-axis. **B** Graph showing normalized calcium activity of the same field following TTX treatment, with thresholds from baseline activity to minimize contribution of background noise to detected signal. **C** Graph showing normalized calcium activity of the same pharmacology sample from **B** with the respective intensity scale (y-axis) and background estimated from the TTX-treated sample, Scale 0–0.397. Scale bar indicates: 60 s on x-axis and 0.02 units of normalized mean fluorescence intensity on y-axis. **D** Boxplots showing the calcium activity of neurons from baseline and TTX-treated fields

Finally, we tested the accuracy of our peak detection methodology by applying tetrodotoxin (TTX), a pharmacological agent that blocks sodium channels thus preventing neuronal activity, to hiPSC-derived neuron cultures. We measured calcium transients in neurons before and after the application of TTX (Fig. 11A, B). In this case, the background intensity or the height threshold used in building Motif correlation maps are estimated based on the baseline, not based on the total data including the manipulation (Fig. 11C). We see a decrease in the number of calcium events per ROI following TTX treatment, when controlling for various covariants in the data (Fig. 11D; mean ± SEM from 7 lines, Baseline 13.83 ± 0.35; TTX 2.84 ± 0.068, linear mixed effects model p-value < 2e-16). This demonstrates that CaPTure is accurately detecting synaptic events.

When all data from a given dataset was processed, we compiled all metrics (Step 7). We used the extracted metrics to make comparisons across different experimental manipulations, and made a custom R script for further analysis, to compare the frequency and type of events between neurons derived from individuals diagnosed with schizophrenia and neurotypical controls as previously described [13].

## Conclusions

Here we have demonstrated the utility of CaPTure to segment neurons and to detect and classify calcium events. CaPTure’s advantages include its ability to effectively segment neurons from surrounding neuropil, which can cause noise in the activity traces and reduce the amplitude and prominence of true events. Additionally, CaPTure uses a rolling average (of 50 frames) intensity normalization (df/f) that effectively estimates the baseline background signal resulting in reduced incidence of false positives in the final data. The workflow allows for parallel processing of data from large studies, without requiring significant user input or parameterizations. The motif-based method for picking events gives users more insight about the data, including the shape and duration of events. Additionally, the acquisition of high resolution images of cultured neurons could allow users to perform machine learning-based classification on neurons or traces. Calcium events are considered a proxy for neuronal activity, and thus CaPTure provides a powerful tool for researchers to make assessments about the relative cellular and ensemble activity of neurons in culture.

## Availability and requirements

Project name: CapTure.

Project home page: https://github.com/LieberInstitute/CaPTure

Operating system(s): MAC, Windows, linux.

Programming language: MATLAB.

Other requirements: MATLAB image processing toolbox, version 2019a or newer.

License: GNU GENERAL PUBLIC LICENSE, Version 3, 29 June 2007.

Any restrictions to use by non-academics: license required.

## Supplementary Information

**Additional file 1: Figure S1. **Motif shapes: These plots show the shapes of 23 motifs used in the CaPTure workflow. Motifs 1–16 are adapted from the FluoroSNNAP software and motifs 17–23 were generated based on our data.

## Data Availability

The datasets analyzed and code used during the current study are available in the Github repository, https://github.com/LieberInstitute/CaPTure.

## Abbreviations

hiPSC: Human induced Pluripotent Stem Cell
GECI: Genetically Encoded Calcium Indicator
ROI: Region of Interest
TTX: Tetrodotoxin
DFF, df/f: Delta fluorescence/fluorescence
