# Supplementary figures and images for: CaPTure: Calcium PeakToolbox for analysis of in vitro calcium imaging data
Source: BMC Neurosci. 2022 Nov 30;23:71. doi: 10.1186/s12868-022-00751-7 (PMC9710137; doi:10.1186/s12868-022-00751-7)

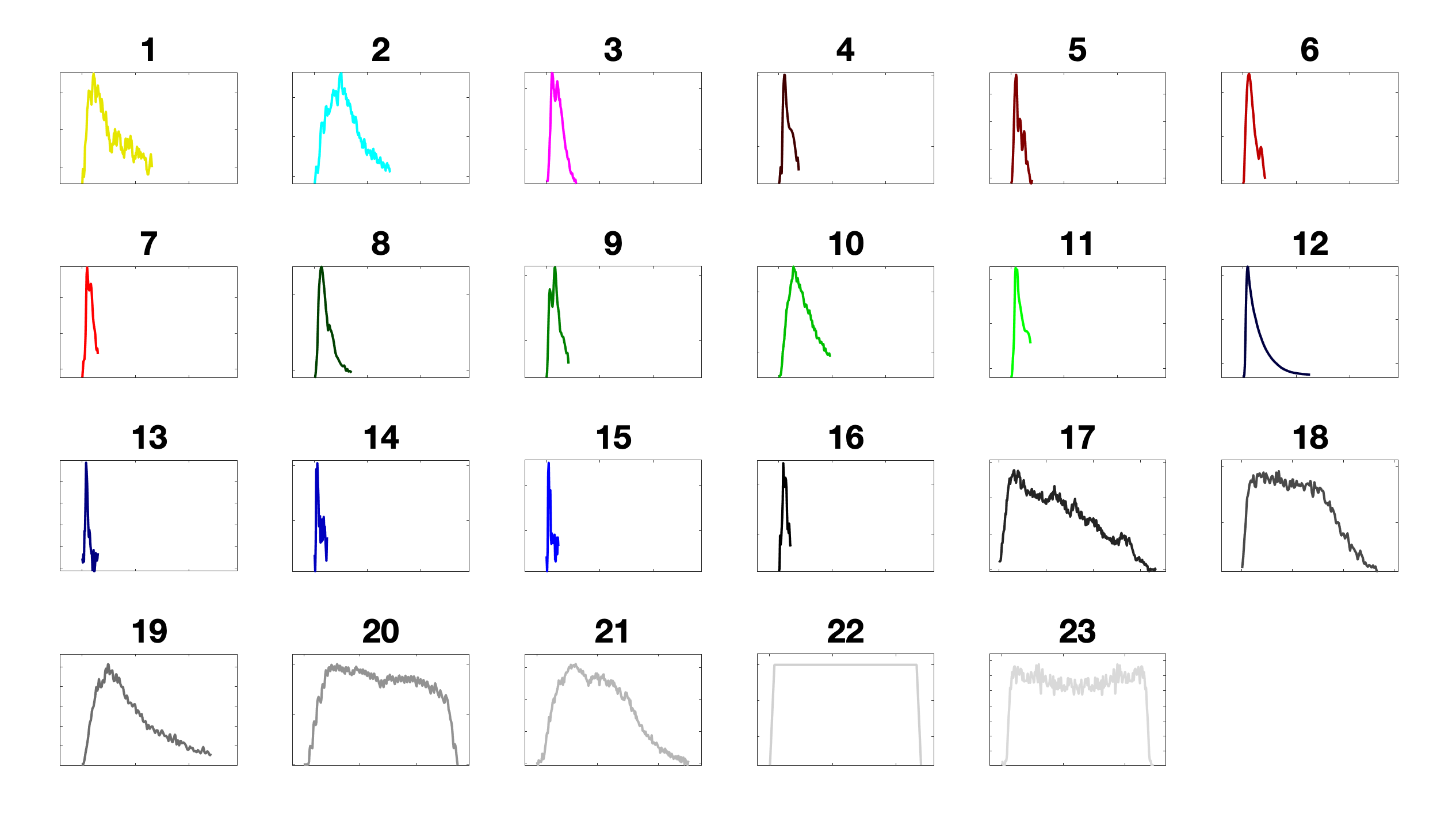

Supplement: Supplementary file 1 — Additional file 1: Figure S1. Motif shapes: These plots show the shapes of 23 motifs used in the CaPTure workflow. Motifs 1–16 are adapted from the FluoroSNNAP software and motifs 17–23 were generated based on our data. [file 12868_2022_751_MOESM1_ESM.png]
